# Concomitant singularities of Yb-valence and magnetism at a critical lattice parameter of icosahedral quasicrystals and approximants

**DOI:** 10.1038/s41598-020-74124-7

**Published:** 2020-10-13

**Authors:** Keiichiro Imura, Hitoshi Yamaoka, Shinjirou Yokota, Kazushi Sakamoto, Yoshiya Yamamoto, Takuma Kawai, Keisuke Namba, Shinnosuke Hirokawa, Kazuhiko Deguchi, Nozomu Hiraoka, Hirofumi Ishii, Jun’ichiro Mizuki, Tsutomu Ishimasa, Noriaki K. Sato

**Affiliations:** 1 Graduate School of Science, Nagoya University, Nagoya, 464-8602 Japan; 2 RIKEN SPring-8 Center, Sayo, Hyogo 679-5148 Japan; 3 Graduate School of Science and Technology, Kwansei Gakuin University, Sanda, 669-1337 Japan; 4 National Synchrotron Radiation Research Center, Hsinchu, 30076 Taiwan; 5 Toyota Physical & Chemical Research Institute, Nagakute, 480-1192 Japan

**Keywords:** Condensed-matter physics, Electronic properties and materials

## Abstract

Non-Fermi-liquid (NFL), a significant deviation from Fermi-liquid theory, usually emerges near an order-disorder phase transition at absolute zero. Recently, a diverging susceptibility toward zero temperature was observed in a quasicrystal (QC). Since an electronic long-range ordering is normally absent in QCs, this anomalous behaviour should be a new type of NFL. Here we study high-resolution partial-fluorescence-yield x-ray absorption spectroscopy on Yb-based intermediate-valence icosahedral QCs and cubic approximant crystals (ACs), some of which are new materials, to unveil the mechanism of the NFL. We find that for both forms of QCs and ACs, there is a critical lattice parameter where Yb-valence and magnetism concomitantly exhibit singularities, suggesting a critical-valence-fluctuation-induced NFL. The present result provides an intriguing structure–property relationship of matter; size of a Tsai-type cluster (that is a common local structure to both forms) tunes the NFL whereas translational symmetry (that is present in ACs but absent in QCs) determines the nature of the NFL against the external/chemical pressure.

## Introduction

Landau’s Fermi-liquid (FL) theory well describes low temperature properties of a metal, such as temperature independent behaviour in the specific heat coefficient *C*/*T* and the uniform magnetic susceptibility $\chi$. In the vicinity of a quantum critical point (QCP), for example, a magnetic QCP where a Néel temperature continuously vanishes to zero, such a FL feature is modified by order-parameter fluctuations coupled with low-energy electron-hole excitations around the Fermi level, into another form of $C/T \propto -T^{1/2}$ and $\chi \propto -T^{1/4}$ in the case of a three-dimensional (3D) case^1^. We note that neither of them diverges as $T \rightarrow 0$. In contrast, Yb-based heavy fermion crystals ($\beta$-$\text{YbAlB}_4$ and $\text{YbRh}_2\text{Si}_2$) and Au–Al–Yb quasicrystal (QC) exhibit the following divergent behaviour toward zero temperature^2–6^,

1 $$\begin{aligned} C/T\ \propto -\ln {T}\quad {{\mathrm{and}}}\quad \chi \ \propto T^{-\zeta } \quad{{\mathrm{with}}}\quad \zeta \ \approx 0.5. \end{aligned}$$

Long-range ferromagnetism has not been observed in these systems. Note that there is no long-range ordered state in QCs; only exception is superconductivity in Al–Zn–Mg QC^7^. The origin of the NFL features expressed above thus remains veiled in mystery^8–11^.

**Figure 1:**
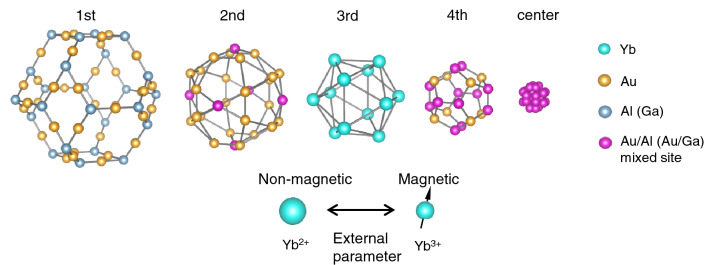
Tsai-type cluster consisting of concentric polyhedral shells in Au–Al–Yb QC and AC. The Tsai-type cluster possesses five concentric polyhedral shells: moving from outside to center, a rhombic triacontahedron (containing 60 atoms), an icosidodecahedron (30 atoms), an icosahedron with 12 Yb atoms, a dodecahedron (20 atoms) and a tetrahedron (4 atoms)^14,15^. The Yb atoms (denoted by light-blue balls in the 3rd shell) are located on the vertices of the icosahedron. Each Yb atom has an IV state between non-magnetic $\text{Yb}^{2+}$ and magnetic $\text{Yb}^{3+}$. Pink balls indicate Au/Al mixed sites. In the Au–Ga–Yb system, for example, Al is assumed to be replaced by Ga.

QCs are metallic alloys that possess long-range quasiperiodic structure with specific diffraction symmetry such as five- or tenfold rotational symmetry that is incompatible with translational symmetry and hence forbidden to periodic crystals^12,13^. Bragg reflection spots in QCs are very sharp like in periodic crystals (see Fig. S1), which is contrasted with diffuse ring in random amorphous solids. Au–Al–Yb QC consists of building blocks named Tsai-type cluster, a set of polyhedrons of decreasing size that are nested like a matryoshka doll (see Fig. 1)^14,15^. Yb atoms are located on the vertices of the icosahedron, and the edge length connecting Yb atoms is approximately 0.55 nm (Ref.^6^). These clusters are aligned over long distances as evidenced by the sharp diffraction spots as mentioned above. This quasiperiodic arrangement of the cluster forms a QC. Interestingly, quasiperiodic structures can be produced by projecting 6D hypercubic lattices onto 3D space^16,17^, meaning that QCs are characterised by 6D lattice parameter $a_{\mathrm{6D}}$.

Approximant crystal (AC) is a periodic phase (space group: $Im{\bar{3}}$) that has a composition close to that of QC. Its building blocks, Tsai-type clusters, form body-centered-cubic (BCC) structure. AC is characterised by conventional 3D lattice parameter $a_{\mathrm{3D}}$, and $a_{\mathrm{3D}}$ is generically related to $a_{\mathrm{6D}}$ of the relevant QC by the equation^18^,

2 $$\begin{aligned} a_{\mathrm{3D}}\simeq 1.946\ a_{\mathrm{6D}}. \end{aligned}$$

Experimentally, AC is used to discriminate the effects of the local and global structure on the physical properties; if some property is observed only in QCs, then its origin should be ascribed to the quasiperiodicity; otherwise, the property can be considered as a characteristic of the common building block, i.e., the icosahedral Tsai-type cluster. Theoretically, on the other hand, AC is useful to compare between calculation and experiment^19^ because any calculation such as a band structure calculation is almost impossible for QCs.

Au–Al–Yb AC also exhibits the NFL phenomena of Eq. (1)^6^. The difference between the QC and the AC appears in the emergence of the divergence in the magnetic susceptibility under pressure; whereas the AC exhibits the divergent feature only at a critical pressure ($P_{\mathrm{c}} \approx 2$ GPa)^20^, the QC shows the divergency in the magnetic susceptibility at any pressure studied thus far, meaning that the NFL of the QC is robust against external pressure^6^. The same exponent $\zeta$ between both forms indicates that the NFL is driven by the same mechanism for the QC and the AC.

The purpose of the present study is to experimentally identify the mechanism of the NFL phenomena observed in the Yb-based QC and AC. Two points should be noted: Yb ion in the Au–Al–Yb QC was experimentally revealed to be in an intermediate valence (IV) state between divalent and trivalent states^21^. (The spatial and temporal fluctuation was confirmed for both the Al–Al–Yb QC and AC from preliminary experiments of photoemission spectroscopy^22^.) Theoretically, a valence fluctuation model predicts the anomalous exponent, $0.5 \le \zeta \le 0.7$, which covers that of Eq. (1)^23^. These motivate us to directly study a valence state of Yb of the Au–Al–Yb QC, using a cutting-edge technique, i.e., a high-resolution partial-fluorescence-yield x-ray absorption spectroscopy (PFY–XAS)^24,25^.

In this study, we have started our investigation from search for new ambient-pressure IV QCs and ACs because the Au–Al–Yb system is the only QC/AC in which an IV state was established at ambient pressure. We have successfully found new Tsai-type QCs/ACs that are in IV state. Unexpectedly, we have found that the Au–Al–Yb QC and AC are both located on the verge of a critical valence transition/crossover. This has twofold implications: First, the result strongly suggests that the NFL is driven by the quantum valence fluctuation. Second, it reveals a novel structure–property relationship of matter; the local structure (probably the cluster size), which determines the bond overlap and (as a result) the Yb mean-valence, plays a crucial role in the emergence of the quantum critical phenomena whereas the global structure (i.e., the periodic or quasiperiodic arrangement of the cluster) determines whether the NFL emerges over a small or wide range of cluster size. To the best of our knowledge, this is the first report of such the relationship. This becomes possible due to the comparative study between the QC and the AC.

## Results

### Sample preparation and characterization

We synthesised three types of (quasi)ternary QC alloys [Au–Ga–Yb, (Au/Cu)–Al–Yb and Au–(Al/Ga)–Yb] and relevant AC alloys (Au–Ga–Yb, Pd–Ga–Yb, Pd–Ge–Yb and Au–Al–Yb); among them, Au–Ga–Yb and Au–(Al/Ga)–Yb systems are a new family of QCs/ACs that have not been reported so far to the best of our knowledge. Here, Au–Ga–Yb QC and (Au/Cu)–Al–Yb QC, for example, denote $\text{Au}_{84-x}\text{Ga}_x\text{Yb}_{16}$ and ($\text{Au}_{1-x}\text{Cu}_x$)$_{49}\text{Al}_{34}\text{Yb}_{17}$, respectively. See Methods and Supplementary Information (SI) for other alloys. Powder x-ray diffraction (XRD) measurements (see Fig. S1), which were conducted at room temperature, showed that samples prepared here have the same pattern as the Tsai-type cluster based Au–Al–Yb QC/AC and (Au/Cu)–Al–Yb QC^15,26^. This suggests that the Tsai-type structure is maintained in the present substituted systems; detailed structure analysis of these (quasi)ternary QCs should be carried out in the future.

### Spectroscopic characterization of IV QCs and ACs

**Figure 2:**
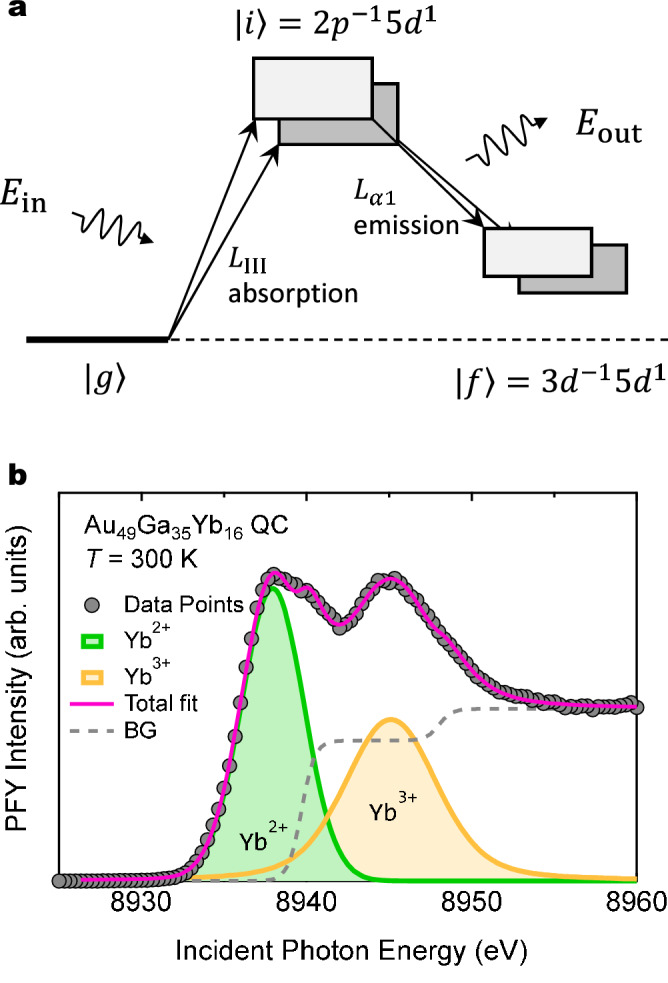
PFY–XAS spectrum of Yb-based IV system. (**a**) Schematic energy diagram of a resonant x-ray emission process of IV Yb ions. $\left. |g \right\rangle$, $\left. |i \right\rangle$ and $\left. |f \right\rangle$ denote the initial, intermediate and final state, respectively. (**b**) PFY spectra of $\text{Au}_{49}\text{Ga}_{35}\text{Yb}_{16}$ QC. The green and orange curves denote the resonant component arising from the $\text{Yb}^{2+}$ and $\text{Yb}^{3+}$ configuration, respectively. The broken line denotes the background (fluorescence) component. The pink solid line indicates summation of the resonant and fluorescence components.

Figure 2a shows a schematic energy scheme of the resonant x-ray emission process for intermediate-valence materials. When an incident x-ray energy $E_{\mathrm{in}}$ is set across to the Yb $L_{\mathrm{3}}$ absorption edge, a resonant absorption occurs and, as a result, 2*p* core electron is excited to 5*d* conduction band, leading to two intermediate states as $\left. |i\right\rangle \ =2p^{-1}{4f}^{13}{5d}^1$ and $2p^{-1}{4f}^{14}{5d}^1$ with different core-hole potentials (called chemical shift) between the two Yb configurations. In a short lifetime of 2*p* core-hole, $L_{\alpha 1}$ emission $3d\rightarrow 2p$ occurs with an energy $E_{\mathrm{out}}$, leading to the final state $\left. |f\right\rangle \ =3d^{-1}{4f}^{13}{5d}^1$ and $\ 3d^{-1}{4f}^{14}{5d}^1$. Correspondingly, the intermediate valence system yields the double-peaked structure as demonstrated for Au–Ga–Yb QC in Fig. 2b. Note that, compared to normal-XAS technique, the PFY-XAS technique yields a higher resolution because only one quantum transition is selected and thus the life-time broadening of the spectra is suppressed^24^.

The spectrum consists of two components, a resonant component yielding the double-peaked structure, and a double-step-like fluorescence component contributing to the background spectrum, as observed in other IV compounds^27,28^. The resonant and fluorescence components were assumed to be described by a double-pseudo-Voight and double-Sigmoid function, respectively. See SI for another choice of functions. The resonant component is decomposed into two components coming from $\text{Yb}^{2+}$ and $\text{Yb}^{3+}$ configuration, each forming a peak at 8938 and 8945.4 eV (denoted by green and orange lines in Fig. 2b), respectively. Using the intensities of the resonant components of the $\text{Yb}^{2+}$ and $\text{Yb}^{3+}$ configurations, $I_2$ and $I_3$, we define an Yb mean-valence $\nu$ as follows^27^,

3 $$\begin{aligned} \nu =\ 2+\frac{I_3}{I_2+I_3}. \end{aligned}$$

Rose-pink line in the figure indicates summation of the resonant and fluorescence components fitted to the data.

### Discovery of new IV QCs/ACs

**Figure 3:**
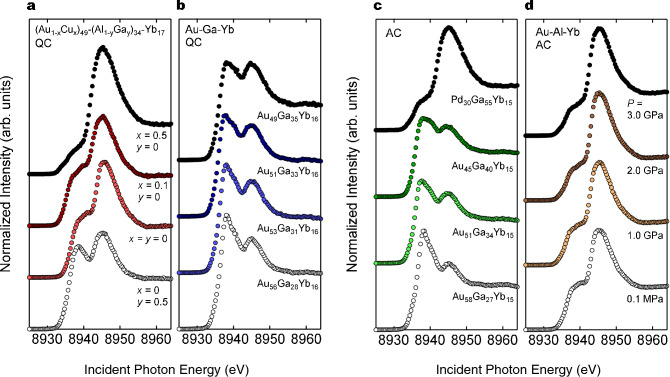
PFY–XAS spectra of Yb-based QCs and ACs measured at 300 K. (**a**) Spectra of ($\text{Au}_{1-x}\text{Cu}_x$)–($\text{Al}_{1-y}\text{Ga}_y$)–Yb QCs: from top to bottom, ($\text{Au}_{0.5}\text{Cu}_{0.5}$)–Al–Yb QC, ($\text{Au}_{0.9}\text{Cu}_{0.1}$)–Al–Yb QC, Au–Al–Yb QC and Au–($\text{Al}_{0.5}\text{Ga}_{0.5}$)–Yb QC. (**b**) Spectra of Au–Ga–Yb QCs with a different Au/Ga composition. (**c**) Spectra of Pd–Ga–Yb AC and Au–Ga–Yb ACs with a different Au/Ga composition. (**d**) Spectra of Au–Al–Yb AC under pressure (*P*) up to 3.0 GPa. According to a magnetization measurement^20^, the NFL emerges at $P \simeq 2$ GPa.

Figure 3a,b show PFY–XAS spectra measured at 300 K for two series of QCs, ($\text{Au}_{1-x}\text{Cu}_x$)–($\text{Al}_{1-y}\text{Ga}_y$)–Yb and Au–Ga–Yb with a different Au/Ga ratio, respectively. The sample denoted “$x=y=0$” in Fig. 3a corresponds to the Au–Al–Yb QC. All spectra clearly show the double-peaked structure, a signature of the IV state. These results inevitably indicate that all the alloys studied here are a new family of IV QCs. Note that the valence ratio $\text{Yb}^{3+}$/$\text{Yb}^{2+}$ remarkably varies with the materials. For comparison, we show PFY–XAS spectra of Pd–Ga–Yb AC and Au–Ga–Yb ACs with a different Au/Ga ratio in Fig. 3c, and the Au–Al–Yb AC under pressure in Fig. 3d. All the spectra exhibit the double-peaked structure signifying the IV state.

We stress that for both forms, the spectral line shape and hence the Yb mean-valence varies drastically with elemental substitutions of Au/Ga, Au/Cu and Al/Ga. A similar variation in PFY spectral shape was observed in heavy-fermion compounds under pressure, which was ascribed to a change in *c*-*f* hybridization strength associated with the pressure-induced lattice-parameter compression^28^. (Here, *c* and *f* indicates conduction and 4*f* electrons, respectively.) By analogy with this, we attribute the spectral-shape change observed here primarily to the hybridization-strength change via the lattice parameter change (see discussion below for more details). Hereafter, the lattice-parameter change caused by the constituent-element substitution is referred to as a chemical pressure effect.

### Valence instability driven by tuning lattice parameter

**Figure 4:**
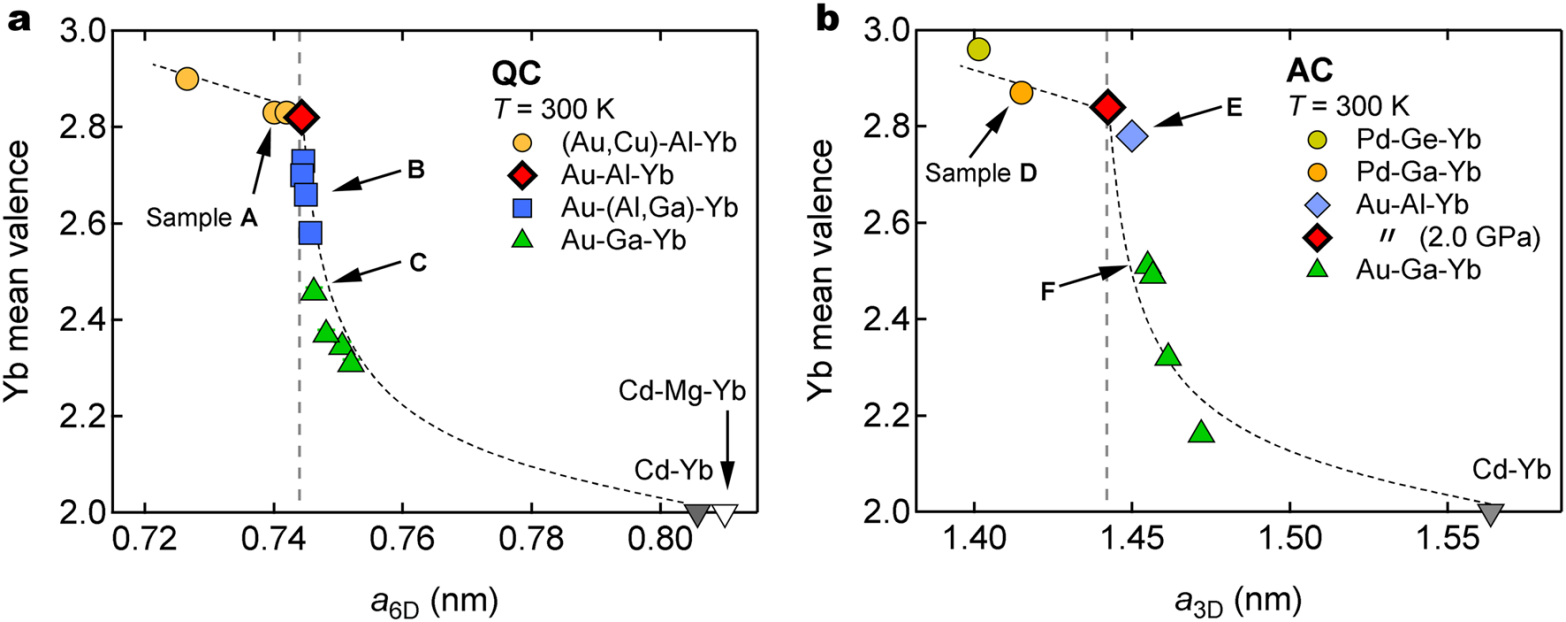
Lattice parameter dependence of Yb mean-valence. Dependences of Yb mean-valence ($\nu$) on 6D lattice parameter ($a_{\mathrm{6D}}$) of the QCs (**a**) and 3D lattice parameter ($a_{\mathrm{3D}}$) of the ACs (**b**). Vertical dashed lines indicate critical lattice parameters $a_{\mathrm{6D}}^{\mathrm{c}}=0.7443 \,\text{nm}$ of Au–Al–Yb QC and $a_{\mathrm{3D}}^{\mathrm{c}}=1.4424 \,\text{nm}$ of Au–Al–Yb AC at $P_{\mathrm{c}} (\simeq 2.0$ GPa), respectively. Dotted curves are guides for the eyes. Sample A-F indicate ($\text{Au}_{0.5}\text{Cu}_{0.1}$)$_{49}\text{Al}_{34}\text{Yb}_{17}$ QC, $\text{Au}_{49}$($\text{Al}_{0.7}\text{Ga}_{0.3}$)$_{34}\text{Yb}_{17}$ QC, $\text{Au}_{49}\text{Ga}_{35}\text{Yb}_{16}$ QC, $\text{Pd}_{30}\text{Ga}_{55}\text{Yb}_{15}$ AC, $\text{Au}_{49}\text{Al}_{36}\text{Yb}_{15}$ AC and $\text{Au}_{45}\text{Ga}_{40}\text{Yb}_{15}$ AC, respectively. Detailed sample information (alloy composition, lattice parameter, etc.) is summarised in Tables S1 and S2. The data points for Cd–Yb and Cd–Mg–Yb QCs (in which Yb is divalent) were taken from Refs.^29^ and^30^.

Figure 4a shows the Yb mean-valence $\nu$ of the IV QCs as a function of the 6D lattice parameter $a_{\mathrm{6D}}$. Surprisingly, all data fall on a single curve as denoted by the broken line. The mean-valence $\nu$ gradually decreases to $\sim 2.8$ as $a_{\mathrm{6D}}$ approaches $a_{\mathrm{6D}}^{\mathrm{c}}=0.7443\,\mathrm{nm}$ (red diamond), the lattice parameter of the Au–Al–Yb QC. Beyond this value, $\nu$ plummets to $\sim 2.5$ and then slowly decreases toward 2; note that Cd–Yb and Cd–Mg–Yb QCs are in the $\text{Yb}^{2+}$ state^29,30^. We stress that the curve slope, $d\nu /da_{\mathrm{6D}}$, changes discontinuously at $a_{\mathrm{6D}}^{\mathrm{c}}$, indicating that there occurs a valence instability such as a transition or crossover at that point. Hereafter, we denote $a_{\mathrm{6D}}^{\mathrm{c}}$ as 6D critical lattice parameter.

Figure 4b shows the Yb mean-valence $\nu$ of the IV ACs as a function of the 3D lattice parameter $a_{\mathrm{3D}}$. We find the similar feature to the QCs shown above, indicating again that the valence instability occurs at $a_{\mathrm{3D}}^{\mathrm{c}}= 1.4424\, {\mathrm{nm}}$, the lattice parameter of the Au–Al–Yb AC at $P_{\mathrm{c}} (\simeq 2\,\text{GPa}$). We denote $a_{\mathrm{3D}}^{\mathrm{c}}$ as 3D critical lattice parameter.

What should be stressed here is the similarity between the QCs and the ACs. This similarity suggests that the presence of the critical lattice parameters is not related to the absence/presence of the periodicity but rather ascribed to the local structure such as the size of the Tsai-type cluster. Structural analysis must be performed to measure the cluster size, but it is natural to assume that the cluster size is proportional to the lattice parameter.

### Magnetic instability at the critical lattice parameter

**Figure 5:**
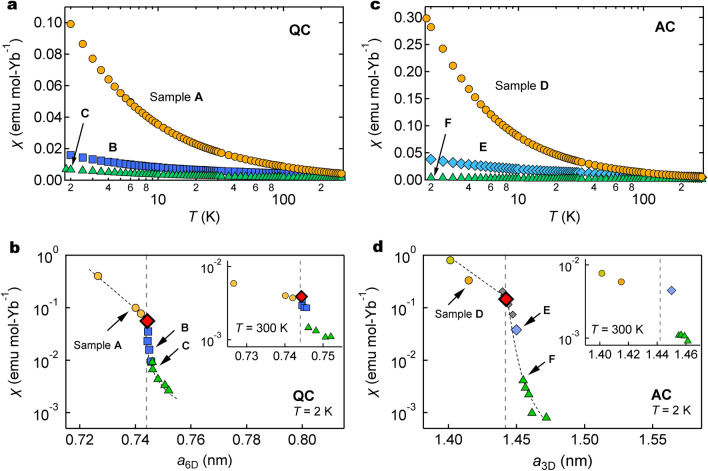
Magnetic susceptibility of Yb-based QCs and ACs. (**a, c**) Temperature dependence of uniform magnetic susceptibility ($\chi$) of representative QCs and ACs (measured at $H = 1000 \,\text{Oe}$). (**b, d**) Dependence of magnetic susceptibility on 6D lattice parameter ($a_{\mathrm{6D}}$) and 3D lattice parameter ($a_{\mathrm{3D}}$) of the QCs and ACs measured at 2 K (and at 300 K, inset). Vertical dashed lines indicate critical lattice parameters $a_{\mathrm{6D}}^{\mathrm{c}}=0.7443\,\text{nm}$ of Au–Al–Yb QC and $a_{\mathrm{3D}}^{\mathrm{c}}=1.4424 \,\text{nm}$ of Au–Al–Yb AC at $P_{\mathrm{c}} (\simeq 2.0$ GPa), respectively. Small gray diamonds show data points of Au–Al–Yb AC (sample E) taken from Ref.^20^. Dotted lines are guide for the eye.

Figure 5a shows the temperature dependence of the uniform magnetic susceptibility $\chi =M/H$ of representative QCs between $\sim 1.8 \,\text{K}$ and room temperature, where *M* and *H* denote magnetization and external magnetic field, respectively. We find that the magnetism strongly varies with alloy composition; sample A shows a strong temperature dependence over a wide temperature range (note a logarithmic scale in the horizontal axis), whereas sample C only shows a weak temperature dependence at low temperatures; note that the low-temperature increase in $\chi (T)$ for both samples is related to the quantum criticality as described below.

Figure 5b shows $a_{\mathrm{6D}}$ dependence of the magnetic susceptibility $\chi$ at $T=2\,\text{K}$ of the QCs; inset shows $a_{\mathrm{6D}}$ dependence of $\chi$ at room temperature. We find that the $\chi$ decreases at a remarkably fast rate (note a logarithmic scale in the vertical axis) on the right side of $a_{\mathrm{6D}}^{\mathrm{c}}$ (diamond).

We stress the close resemblance between Figs. 4a and 5b, indicating that the magnetism is tightly related to the Yb mean-valence anticipated from the fact that $\text{Yb}^{3+}$ is magnetic whereas $\text{Yb}^{2+}$ is nonmagnetic. This indicates that the valence and magnetic instabilities occur simultaneously.

A similar feature is observed for the ACs; see Fig. 5c for the temperature dependence of the uniform magnetic susceptibility $\chi$, and see Fig. 5d for the $a_{\mathrm{3D}}$ dependence of $\chi$ at $T=2$ K and room temperature (inset). The instability of magnetism occurs at $a_{\mathrm{3D}}^{\mathrm{c}}$ (the large red diamond). Again, the similarity between Figs. 4b and 5d indicates the simultaneous instability of Yb valence and magnetism.

The interrelationship observed here between valence, magnetism and lattice parameter is compatible with the pressure effect for Yb-based heavy fermions; when a non-magnetic Yb-based heavy fermion is pressurized and, as a result, the lattice shrinks, a magnetic order usually appears due to the emergence of the magnetic $\text{Yb}^{3+}$ ions with a smaller ionic radius.

Figure 5b,d shows the close resemblance between the QCs and the ACs; when $a_{\mathrm{6D}}$ $(a_{\mathrm{3D}})$ is very slightly increased above $a_{\mathrm{6D}}^{\mathrm{c}}$ ($a_{\mathrm{3D}}^{\mathrm{c}}$), the $\chi$ suddenly drops by one order of magnitude at $T=2$ K. Similar to the valence instability, this resemblance suggests that the magnetic instability is not related to the absence/presence of the periodicity but rather ascribed to a change in the local structure such as the size of the Tsai-type cluster.

### NFL behaviour at the critical lattice parameters

In Figs. 4 and 5, the QCs/ACs can be separated into two groups; the strong magnetism group (including QC sample A and AC sample D) that has a smaller lattice parameter than $a_{\mathrm{6D/3D}}^{\mathrm{c}}$ and a Yb mean-valence close to 3+, and the weak magnetism group (including QC sample C and AC sample F) that has a larger lattice parameter than $a_{\mathrm{6D/3D}}^{\mathrm{c}}$ and a Yb mean-valence rather close to 2+. Note that the critical lattice parameters $a_{\mathrm{6D}}^{\mathrm{c}}$ and $a_{\mathrm{3D}}^{\mathrm{c}}$ satisfy Eq. (2). These two groups are contiguous, and the Au–Al–Yb QC and the Au–Al–Yb AC at $P_{\mathrm{c}}$ are on the border of them.

**Figure 6:**
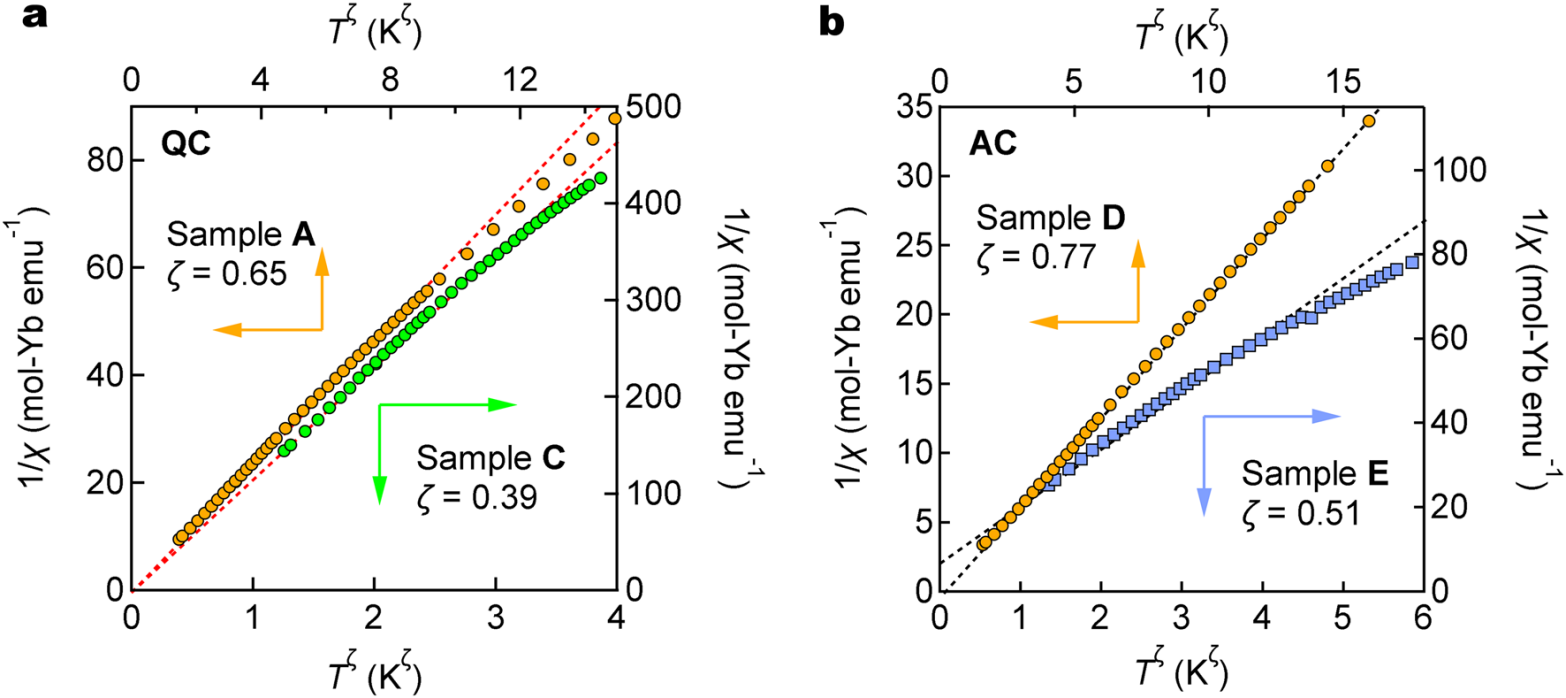
Inverse magnetic susceptibility of Yb-based QCs and ACs. $T^{\zeta }$ dependence of inverse magnetic susceptibility ($1/\chi$) of the QCs (**a**) and ACs (**b**). The critical exponent $\zeta$ is given in the figure. Dotted lines indicate extrapolation to absolute zero. When the extrapolation goes through the origin of the figure, the magnetic susceptibility diverges toward zero temperature.

Figure 6a shows the inverse magnetic susceptibility $1/\chi$ of the QCs as a function of $T^\zeta$, where $\zeta =0.65$ and 0.39 for sample A and C, respectively. Note that both samples are away from the critical lattice parameter $a_{\mathrm{6D}}^{\mathrm{c}}$ (see Figs. 4 and 5), nevertheless, they seem to exhibit the divergent feature within the temperature range measured here; the extrapolation toward zero temperature (the broken lines) goes through the origin of the figure. (The divergence should be confirmed by further experiments down to lower temperatures.) This result against chemical pressure corresponds to the robustness of the NFL feature against external pressure^6^.

Figure 6b shows the results of the ACs with $\zeta =0.77$ and 0.51 for sample D and E, respectively. These samples are away from the critical lattice parameter $a_{\mathrm{3D}}^{\mathrm{c}}$ (see Figs. 4 and 5), and they do not show the divergence of the susceptibility: in contrast to the QCs shown above, extrapolation toward zero temperature (the broken lines) does not go through the origin of the figure within the measured temperature region. Again, this result of the chemical pressure effect is similar to the external pressure effect; the magnetic susceptibility of the Au–Al–Yb AC shows the divergence only at $P=P_{\mathrm{c}}$.

In summary, whereas the ACs exhibit the unusual temperature dependence of the magnetic susceptibility at the critical lattice parameter, the QCs show the anomalous feature of the susceptibility over a range of the lattice parameter at both sides of the critical lattice parameter. This difference in the nature of the NFL against the chemical pressure between the QCs and the ACs should be ascribed to the difference in the global structure between them, i.e., the quasiperiodic or periodic arrangement of the Tsai-type cluster.

## Discussion

**Figure 7:**
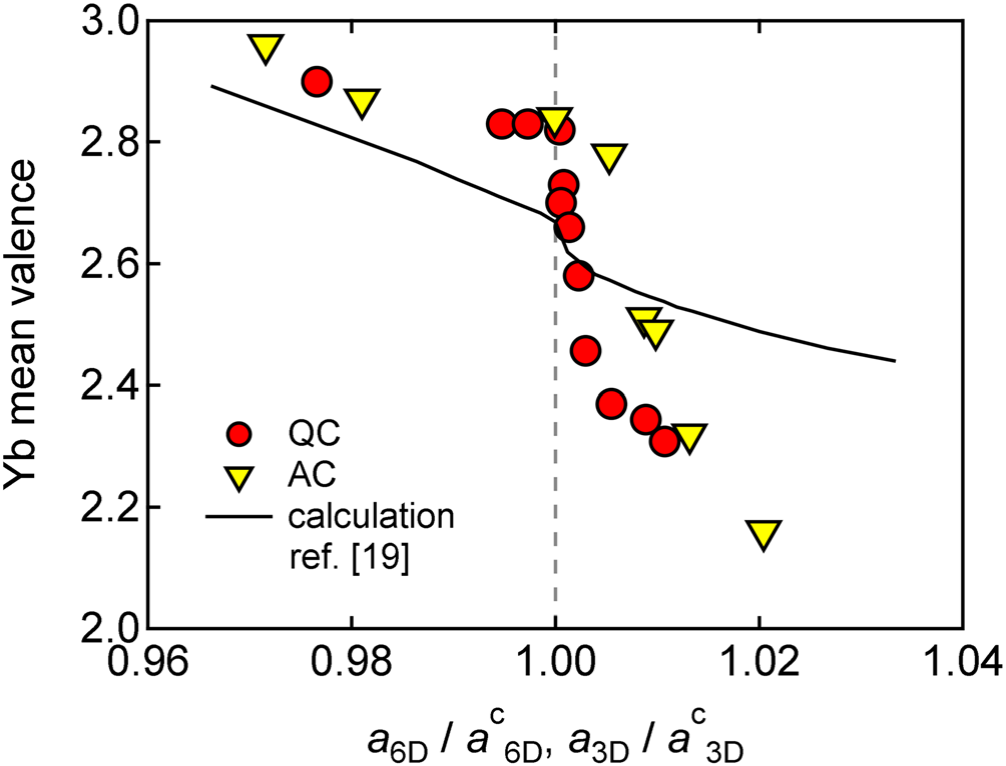
Yb mean-valence as functions of 3D and 6D lattice parameters of Yb-based QCs and ACs. The lattice parameter is normalized with respect to the critical lattice parameter, $a_{\mathrm{6D}}^{\mathrm{c}}$ or $a_{\mathrm{3D}}^{\mathrm{c}}$. The solid line indicates a calculation for the Au–Al–Yb AC by Watanabe and Miyake^19^. The circle and triangle denote the ACs and QCs, respectively. The broken line marks the critical lattice parameter, $a_{\mathrm{6D}}^{\mathrm{c}}$ or $a_{\mathrm{3D}}^{\mathrm{c}}$. Note that the steep slope $d \nu /d a_{\mathrm{3D}}$ just above the critical lattice parameter reflects the divergence of the critical valence fluctuation susceptibility according to Ref.^19^.

Figure 7 summarizes the Yb mean-valence of the Yb-based QCs and ACs as functions of the reduced lattice parameter, $a_{\mathrm{6D}}/a_{\mathrm{6D}}^{\mathrm{c}}$ and $a_{\mathrm{3D}}/a_{\mathrm{3D}}^{\mathrm{c}}$, respectively, demonstrating the close resemblance between the QCs and the ACs. This result leads us to suggest that the Yb mean-valence anomaly at $a_{\mathrm{6D}}/a_{\mathrm{6D}}^{\mathrm{c}}=1$ ($a_{\mathrm{3D}}/a_{\mathrm{3D}}^{\mathrm{c}}=1$) is due to the local structure such as the cluster size, rather than the difference in the global structural property between the QCs and the ACs. In the following, we discuss the possibility that this anomaly emerges as a result of the quantum critical valence fluctuation.

Watanabe & Miyake calculated the relation between $a_{\mathrm{3D}}$ and 4*f* hole number $n_{4f}$ of the Au–Al–Yb AC^19^. We plot the calculated result by the solid line in Fig. 7, assuming the correspondence between $n_{4f}$ and the Yb mean-valence $\nu$ such as $n_{4f}=0 \leftrightarrow \nu =2$ and $n_{4f}=1 \leftrightarrow \nu =3$. The calculation reproduces the observed overall feature rather well, especially a discontinuous feature in $d \nu /d a_{\mathrm{3D}}$ at the critical lattice parameter. (We do not know if there is another theory that quantitatively reproduces the $a_{\mathrm{3D}}$ vs $n_{4f}$ curve to the best of our knowledge.) According to the theory, the slope $d \nu /d a_{\mathrm{3D}}$ corresponds to the critical valence fluctuation susceptibility^19^. Thus, the observed steep slope of $d \nu /d a_{\mathrm{3D}}$ just above $a_{\mathrm{3D}}^{\mathrm{c}}$ means the diverging valence fluctuation susceptibility. This observation leads us to conclude that the quantum critical valence fluctuation is most likely responsible for the NFL behaviour in the Au–Al–Yb AC. The NFL of the Yb-based QCs should also be driven by the same mechanism as mentioned in Introduction.

Despite the semiquantitative agreement between the calculation and the experiment as mentioned above, one may find a rather poor quantitative agreement between them. There are two possible origins of the disagreement: (i) The calculation was made at zero temperature while the experiment was conducted at room temperature. (ii) Whereas the constituent element was changed in the experiment, only the lattice parameter was changed in the calculation: in other words, the experiment and the calculation correspond to the chemical and external pressure effect, respectively.

Generically, the appearance/disappearance of the localized magnetic moment in a metal may be determined by the relative magnitude of the Coulomb repulsion and the mixing energy $\Delta = \pi |V^2| \rho (E_{\mathrm{F}})$, or equivalently the ratio $|\varepsilon _f - E_{\mathrm{F}}|/\Delta$, where *V* is a mixing matrix element between 4*f*- and conduction-electron wave functions, $\rho (E_{\mathrm{F}})$ is the density of states at the Fermi energy $E_{\mathrm{F}}$, and $\varepsilon _f$ is the energy level of 4*f* electron/hole^31^. Note that the smaller the ratio the closer the Yb valence to 2+. Our result (Fig. 7) shows that $|\varepsilon _f - E_{\mathrm{F}}|$ decreases much faster than $\Delta$ with expanding the lattice.

As mentioned in Introduction, the unusual quantum criticality was found in Yb-based heavy fermions; $C/T \propto -\ln {T}$ and $\chi \propto T^{-\zeta }$ with $\zeta = 0.6$ and 0.5 in $\text{YbRh}_2\text{Si}_2$ and $\beta$-$\text{YbAlB}_4$, respectively^2–5^. Such the critical feature similar to the present system allows us to conjecture that the same mechanism is applicable to these strongly correlated materials. (Note here that the critical exponent predicted by the quantum critical valence fluctuation theory ranges from $\sim 0.5$ to $\sim 0.7$^23^.) This conjecture may be supported by a recent observation of the valence crossover in a chemical substitution system of $\alpha$-$\text{YbAlB}_4$^32^. We hope that the present study stimulates a further experiment to get information on the Yb mean-valence of these heavy fermions.

## Methods

### Sample synthesis

We synthesised polycrystalline QC samples [$\text{Au}_{84-x}\text{Ga}_x\text{Yb}_{16}$ ($x = 28$, 31, 33, 35 and 37.3), ($\text{Au}_{1-x}\text{Cu}_x$)$_{49}\text{Al}_{34}\text{Yb}_{17}$ ($x = 0.05$, 0.1 and 0.5) and $\text{Au}_{49}$($\text{Al}_{1-y}\text{Ga}_y$)$_{34}\text{Yb}_{17}$ ($y = 0$, 0.1, 0.2, 0.3 and 0.5)] and polycrystalline AC samples [Au–Ga–Yb alloys with slightly different compositions from the above-mentioned QCs, $\text{Au}_{85-x}\text{Ga}_{x}\text{Yb}_{15}$ ($x = 29$, 34, 36, 38.3 and 40) and $\text{Pd}_{30}\text{Ga}_{55}\text{Yb}_{15}$] using a tetra-arc furnace. The constituent elements (4N-purity Au, 6N-purity Cu, 4N-purity Al, 5N-purity Ga, 3N-purity Pd, 6N-purity Ga and 3N-purity Yb) were placed on a water-cooled Cu hearth in the arc furnace and melted in a high-purity (6N-purity) Ar gas atmosphere. Starting materials of $\text{Pd}_{45.6}\text{Ge}_{38.9}\text{Yb}_{15.5}$ (5N-purity Ge) AC were melted and cooled slowly in an alumina crucible encapsulated by a quartz ampoule under argon atmosphere. The samples studied here are summarised in Tables S1 and S2.

### XRD measurements

To confirm monophasic synthesis, we carried out powder x-ray diffraction (XRD) measurements using a laboratory XRD system (Rigaku, MiniFlex 600). Data collection was carried out at room temperature using the Cu $K_\alpha$ radiation ($\lambda =0.15418 \,\text{nm}$). Within the experimental accuracy, we found no indication of two-phase formation. Details of evaluating the 6D lattice constant are described in SI. The XRD spectra and the resulting 6D/3D lattice parameters are shown in Figs. S1 and S2, respectively.

The powder XRD measurements on Au–Al–Yb QC under pressure were carried out at BL12B2 in SPring-8. For calibration of experimental conditions, we measured $\text{CeO}_2$ as a standard sample. The calibrated incident x-ray energy and distance between sample and 2D imaging-plate were 18.09 keV ($\lambda =0.068563\,\text{nm}$) and 366.06 mm, respectively. X-ray irradiation time was set to 10 minutes. The external pressure was generated by a 3-pin diamond-anvil cell (DAC) with a culet size of 0.3 mm in diameter. Glycerine was selected as a pressure transmitting medium, and the applied pressure was estimated from measurement of the shift of the ruby R1 fluorescence line^33,34^. 1D XRD spectra were obtained by integrating 2D data. The selected XRD patterns are shown in Fig. S3. We found no indication of the two-phase formation within the experimental accuracy.

### PFY-XAS measurements

The PFY–XAS experiment was carried out at the BL12XU inelastic x-ray scattering end-station installed at SPring-8. The monochromatic synchrotron radiation was obtained using a liquid nitrogen-cooled Si (111) double-crystal spectrometer. The $L_{\alpha 1}$ partial emission line of Yb atom was segregated from the total emission line through a Si (620) analyser crystal placed at the Rowland radius of approximately 1.0 m. This technique is able to obtain a higher-resolution spectrum than normal-XAS technique in which all x-ray emission lines, transmitted photons and total photoelectron currents are measured. The measurement temperature was set to 300 K. The external pressure was generated by a He-gas controlled DAC with a culet size of 0.3 mm in diameter. The pressure transmitting medium was Daphne oil 7474, and the pressure was estimated from the shift of the ruby R1 fluorescence line. During the pressurised experiment, the spot was focused using a Kirkpatric-Baez mirror. The measured spectra were analysed as described in the main text and the SI.

### Magnetic susceptibility measurements

The DC magnetic susceptibility was measured using a commercial magnetometer (Quantum Design, MPMS) over a temperature range of 1.8–300 K in a magnetic field of 1000 Oe.

## Supplementary information

Supplementary information

## Data Availability

The data that support the plots within this paper are available from the corresponding author upon reasonable request.

## References

[1] Stewart GR (2001). Non-Fermi-liquid behavior in $$d$$- and $$f$$-electron metals. Rev. Mod. Phys..

[2] Trovarelli O, Geibel C, Mederle S, Langhammer C, Grosche FM, Gegenwart P, Lang M, Sparn G, Steglich F (2000). $$\text{YbRh}_2\text{ Si}_2$$: pronounced non-fermi-liquid effects above a low-lying magnetic phase transition. Phys. Rev. Lett..

[3] Custers J, Gegenwart P, Wilhelm H, Neumaier K, Tokiya Y, Trovarelli O, Geibel C, Steglich F, Pépin C, Coleman P (2003). The break-up of heavy electrons at a quantum critical point. Nature.

[4] Nakatsuji S, Kuga K, Machida Y, Tayama T, Sakakibara T, Karaki Y, Ishimoto H, Yonezawa S, Maeno Y, Pearson E, Lonzarich GG, Balicas L, Lee H, Fisk Z (2008). Superconductivity and quantum criticality in the heavy-fermion system $$\beta $$-$$\text{ YbAlB}_4$$. Nature Phys..

[5] Matsumoto Y, Nakatsuji S, Kuga K, Karaki Y, Horie N, Shimura Y, Sakakibara T, Nevidomskyy AH, Coleman P (2011). Quantum criticality without tuning in the mixed valence compound $$\beta $$-$$\text{ YbAlB}_4$$. Science.

[6] Deguchi K, Matsukawa S, Sato NK, Hattori T, Ishida K, Takakura H, Ishimasa T (2012). Quantum critical state in a magnetic quasicrystal. Nat. Mater..

[7] Kamiya K, Takeuchi T, Kabeya N, Wada N, Ishimasa T, Ochiai A, Deguchi K, Imura K, Sato NK (2018). Discovery of superconductivity in quasicrystal. Nat. Commun..

[8] Watanabe S, Miyake K (2013). Robustness of quantum criticality of valence fluctuations. J. Phys. Soc. Jpn..

[9] Shaginyan VR, Msezane AZ, Popov KG, Japaridze GS, Khodel VA (2013). Common quantum phase transition in quasicrystals and heavy-fermion metals. Phys. Rev. B.

[10] Andrade EC, Jagannathan A, Miranda E, Vojta M, Dobrosavljević V (2015). Non-fermi-liquid behavior in metallic quasicrystals with local magnetic moments. Phys. Rev. Lett..

[11] Otsuki J, Kusunose H (2016). Distributed hybridization model for quantum critical behavior in magnetic quasicrystals. J. Phys. Soc. Jpn..

[12] Shechtman D, Blech I, Gratias D, Cahn JW (1984). Metallic phase with long-range orientational order and no translational symmetry. Phys. Rev. Lett..

[13] Levine D, Steinhardt PJ (1984). Quasicrystals: a new class of ordered structures. Phys. Rev. Lett..

[14] Tsai AP, Guo JQ, Abe E, Takakura H, Sato TJ (2000). A stable binary quasicrystal. Nature.

[15] Ishimasa T, Tanaka Y, Kashimoto S (2011). Icosahedral quasicrystal and 1/1 cubic approximant in AuAlYb alloys. Philos. Mag..

[16] Duneau M, Katz A (1985). Quasiperiodic patterns. Phys. Rev. Lett..

[17] Kalugin PA, Kitaev AY, Levitov LS (1985). $$\text{ Al}_{0.86}\text{ Mn}_{0.14}$$: a six-dimensional crystal. JETP Lett..

[18] Elser V, Henley CL (1985). Crystal and quasicrystal structures in Al–Mn–Si alloys. Phys. Rev. Lett..

[19] Watanabe S, Miyake K (2018). Effects of crystalline electronic field and onsite interorbital interaction in Yb-based quasicrystal and approximant crystal. J. Phys.: Condens. Matter.

[20] Matsukawa S, Deguchi K, Imura K, Ishimasa T, Sato NK (2016). Pressure-driven quantum criticality and $$T$$/$$H$$ scaling in the icosahedral Au–Al–Yb approximant. J. Phys. Soc. Jpn..

[21] Watanuki T, Kashimoto S, Kawana D, Yamazaki T, Machida A, Tanaka Y, Sato TJ (2012). Intermediate-valence icosahedral Au-Al-Yb quasicrystal. Phys. Rev. B.

[22] Matsunami, M. Photoemission spectroscopy of quasicrystals and approximants with f electrons. The Physical Society of Japan 2016 Annual Meeting, 21pBR-7 (ISSN 2189-0803).

[23] Watanabe S, Miyake K (2012). New universality class of quantum criticality in Ce- and Yb-based heavy fermions. J. Phys.: Condens. Matter.

[24] Hämäläinen K, Siddons DP, Hastings JB, Berman LE (1991). Elimination of the inner-shell lifetime broadening in X-ray-absorption spectroscopy. Phys. Rev. Lett..

[25] Rueff JP, Shukla A (2010). Inelastic X-ray scattering by electronic excitations under high pressure. Rev. Mod. Phys..

[26] Oki S, Hiroto T, Muro Y, Tamura R (2014). Magnetic properties of icosahedral (Au, Cu)–Al–Yb quasicrystals. Acta Phys. Pol. A.

[27] Sato H, Yamaoka H, Utsumi Y, Nagata H, Avila MA, Ribeiro RA, Umeo K, Takabatake T, Zekko Y, Mizuki J, Lin J, Hiraoka N, Ishii H, Tsuei K, Namatame H, Taniguchi M (2014). Pressure-induced valence change of YbNiGe$$_3$$ investigated by resonant X-ray emission spectroscopy at the Yb L3 edge. Phys. Rev. B.

[28] Jarrige I, Yamaoka H, Rueff J-P, Lin J-F, Taguchi M, Hiraoka N, Ishii H, Tsuei KD, Imura K, Matsumura T, Ociai A, Kotani A (2013). Unified understanding of the valence transition in the rare-earth monochalcogenides under pressure. Phys. Rev. B.

[29] Kawana D, Watanuki T, Machida A, Shobu T, Aoki K (2010). Intermediate-valence quasicrystal of a Cd–Yb alloy under pressure. Phys. Rev. B.

[30] Watanuki T, Kawana D, Machida A, Tsai AP (2011). Pressure-induced formation of intermediate-valence quasicrystalline system in a Cd–Mg–Yb alloy. Phys. Rev. B.

[31] Matsukawa S, Tanaka K, Nakayama M, Deguchi K, Imura K, Takakura H, Kashimoto S, Ishimasa T, Sato NK (2014). Valence change driven by constituent element substitution in the mixed-valence quasicrystal and approximant Au–Al–Yb. J. Phys. Soc. Jpn..

[32] Kuga K, Matsumoto Y, Okawa M, Suzuki S, Tomita T, Sone K, Shimura Y, Sakakibara T, Nishio-Hamane D, Karaki Y, Takata Y, Matsunami M, Eguchi R, Taguchi M, Chainani A, Shin S, Tamasaku K, Nishino Y, Yabashi M, Ishikawa T, Nakatsuji S (2018). Quantum valence criticality in a correlated metal. Sci. Adv..

[33] Mao HK, Bell PM, Shaner JW, Steinberg DJ (1978). Specific volume measurements of Cu, Mo, Pd, and Ag and calibration of the ruby R1 fluorescence pressure gauge from 0.06 to 1 Mbar. J. Appl. Phys..

[34] Yamaoka H, Zekko Y, Jarrige I, Lin J-F, Hiraoka N, Ishii H, Tsuei K-D, Mizuki J (2012). Ruby pressure scale in a low-temperature diamond anvil cell. J. Appl. Phys..

