# Supplementary material for: Concomitant singularities of Yb-valence and magnetism at a critical lattice parameter of icosahedral quasicrystals and approximants
Source: Sci Rep. 2020 Oct 13;10:17116. doi: 10.1038/s41598-020-74124-7 (PMC7553930; doi:10.1038/s41598-020-74124-7)
Supplement: Supplementary file 1 — Supplementary information [file 41598_2020_74124_MOESM1_ESM.pdf]

Supplementary Information

Concomitant singularities of Yb-valence and magnetism at a critical lattice parameter of icosahedral quasicrystals and approximants

Keiichiro Imura[1]*, Hitoshi Yamaoka[2], Shinjirou Yokota[1], Kazushi Sakamoto[1], Yoshiya Yamamoto[3], Takuma Kawai[3], Keisuke Namba[1], Shinnosuke Hirokawa[1], Kazuhiko Deguchi[1], Nozomu Hiraoka[4], Hirofumi Ishii[4], Jun'ichiro Mizuki[3], Tsutomu Ishimasa[5] & Noriaki K. Sato[1]

1 Graduate School of Science, Nagoya University, Nagoya 464-8602, Japan

2 RIKEN SPring-8 Center, Sayo, Hyogo 679-5148, Japan

3 Graduate School of Science and Technology, Kwansei Gakuin University, Sanda 669-1337, Japan

4 National Synchrotron Radiation Research Center, Hsinchu 30076, Taiwan

5 Toyota Physical & Chemical Research Institute, Nagakute 480-1192, Japan

Figure S1 shows the powder x-ray diffraction (XRD) patterns of the representative quasicrystals (QCs) and approximant crystals (ACs) studied here. Following the structure analysis given in Ref. S1, we indexed almost all diffraction peaks using a set of six and three integers for the QC and AC phase, respectively. Arrows in spectra of $(Au_{0.5}Cu_{0.5})_{49}Al_{34}Yb_{17}$

and $Au_{49}(Al_{0.7}Ga_{0.3})_{34}Yb_{17}$ QC mark unknown phases.

The six-dimensional (6D) lattice parameter ($a_{6\mathrm{D}}$) of the QCs was evaluated by using 6D Elser indices n_i (i = 1–6)[S2-4]. First, we calculated the lattice spacing d in terms of the index obtained above, and then evaluated $a_{6\mathrm{D}}$ for each peak using the following formula:

$$d = \frac{a_{6\mathrm{D}}}{|\sum_{i=1}^{6} n_i \mathbf{e}_{i//}|}, \qquad (1)$$

where the vectors $\mathbf{e}_{i//}$ are those connecting from the center of icosahedron to the 6 vertices with the length $1/\sqrt{2}$. Note that the parameter $a_{6\mathrm{D}}$ is related to the parameter a in Ref. S2 as $a_{6\mathrm{D}} = \sqrt{2}a$. Finally, we evaluated the averaged $a_{6\mathrm{D}}$ by means of the Nelson-Riley's extrapolation method. The diffraction angles used in this study are in the range of approximately $20° < 2\theta < 80°$. For the $Au_{49}Al_{34}Yb_{17}$ QC, for example, we obtained $a_{6\mathrm{D}} =$ 0.7443 nm, consistent with the previous report[S1].

Figures S2a-c show the 6D lattice parameter $a_{6\mathrm{D}}$ and the Yb mean-valence ν of the QCs as a function of the constituent element compositions. We also evaluated Yb mean-valence ν by using the fitting functions dictated in the main text. These data yield the relation between $a_{6\mathrm{D}}$ ($a_{3\mathrm{D}}$) and ν (with the composition as an implicit parameter) shown in Figs. 4a and 4b in the main text. Atomic compositions and lattice parameters (including Yb mean-valence and magnetic susceptibility mentioned below) of all samples studied here are summarised in Tables S1 and S2.

Figure S3a shows the pressure evolution of the Bragg peak profile of $11\bar{1}\bar{1}12$ and $01\bar{1}\bar{2}02$ reflections of Au–Al–Yb QC measured at 300 K under pressure. The two-peak structure characteristic of QC phase was clearly observed in the pressure range up to 4.6 GPa. This suggests that the QC phase can be stable under pressure. Note that if 1/1AC phase exists

in the sample, a peak should be present between these two peaks: no such peak is observed.

We evaluated the lattice spacing d and the normalized lattice spacing $d/d_0\,(P)$ under pressure, where d_0 is d-value at ambient pressure. In Fig. S3b, we show the pressure dependence of the normalized volume $V/V_0\,(P)$, where $V/V_0 = \overline{(d/d_0)}^3$. Assuming the relation $P = B(1 - V/V_0)$ between 0.1 MPa ang 4.6 GPa, we estimated the bulk modulus $B = 127$ GPa, which is comparable to that of other QCs[S5]. For the Au–Al–Yb AC, we have no data of the Bragg peak profile under pressure, and then we evaluated $a_{3\mathrm{D}}(P = 1.96\ \mathrm{GPa}) = \sqrt[3]{V/V_0} \cdot a_{3\mathrm{D}}(P = 0) = 1.4425\ \mathrm{nm}$ using the B value obtained above for the QC.

Figure S4a shows the PFY spectrum of Au–Al–Yb QC measured at 300 K. In the main text, we assumed double-pseudo-Voight and double-Sigmoid functions (Fig. 2b) for the resonant and fluorescence component, respectively. Here, we try another set of functions, that is double-Gaussian and single-sigmoid functions for the resonant and fluorescence component, respectively. This leads to the lattice parameter dependence of the Yb mean-valence ν of the QCs and ACs in Figs. S4b and S4c, respectively. Comparing these with the results in the main text, we find a small difference in the absolute value of ν, but no serious change in the overall feature, meaning that the presence of the valence change/crossover at the critical 3D/6D lattice parameters is not affected by the choice of the fitting functions.

Figure S5 shows the $T^{0.5}$ dependence of the inverse ac magnetic susceptibility of Au–Al–Yb AC at 1.96 GPa in a temperature range between 85 mK and 2 K. The data were taken from Ref. S6. In contrast to the ambient-pressure result shown in Fig. 6b in the main text, the $1/\chi$ vs $T^{0.5}$ curve seems to pass through the origin of the figure, meaning the

divergence of $\chi(T)$ as $T \to 0$.

References

[S1] Ishimasa, T., Tanaka, Y. & Kashimoto, S. Icosahedral quasicrystal and 1/1 cubic approximant in Au–Al–Yb alloys. *Phil. Mag.* **91**, 4218-4229 (2011).

[S2] Elser, V. Indexing problems in quasicrystal diffraction. *Phys. Rev.* B **32**, 4892 (1985).

[S3] Elser, V. The diffraction pattern of projected structures. *Acta Cryst.* A **42**, 36 (1986).

[S4] Kaneko, Y., Arichika, Y. & Ishimasa, T. Icosahedral quasicrystal in annealed Zn–Mg–Sc alloys. *Phil. Mag. Lett.* **81**, 777 (2001).

[S5] Krauss, G., Gu, Q. F., Katrych, S. & Steurer, W. In situ study of icosahedral Zn–Mg–Dy and Co-rich decagonal Al–Co–Ni at high pressures and high temperatures. *J. Phys.: Condens. Matter* **19**, 116203 (2007).

[S6] Matsukawa, S., Deguchi, K., Imura, K., Ishimasa, T. & Sato, N. K. Pressure-Driven Quantum Criticality and *T*/*H* Scaling in the Icosahedral Au–Al–Yb Approximant. *J. Phys. Soc. Jpn.* **85**, 063706 (2016).

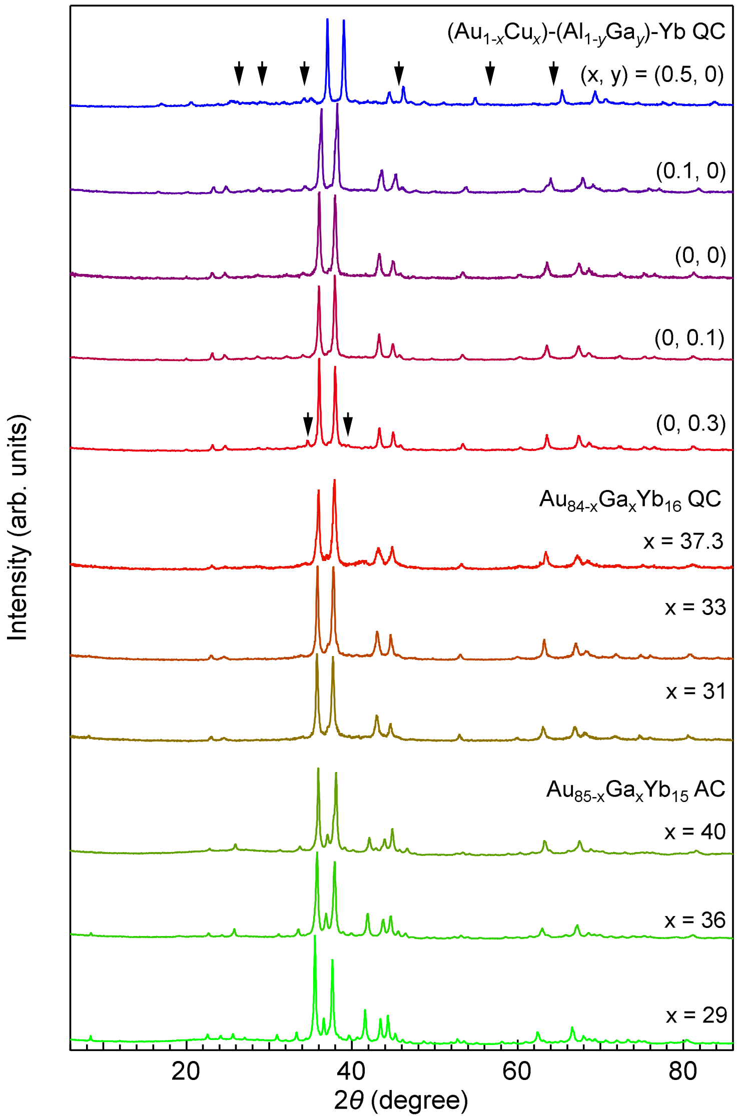

Figure S1 | Powder x-ray diffraction (XRD) spectra of quasicrystals and approximant crystals. Selected powder XRD spectra of $(Au_{1-x}Cu_x)_{49}(Al_{1-y}Ga_y)_{34}Yb_{17}$ QCs with (x, y) = (0.5, 0), (0.1, 0), (0, 0), (0, 0.1) and (0, 0.3), $Au_{84-x}Ga_xYb_{16}$ QCs with $x = 46.7$, 51 and 53 and $Au_{85-x}Ga_xYb_{15}$ ACs with $x = 29$, 36 and 40 are shown. The spectra are vertically shifted for clarity. The arrows show unknown phases.

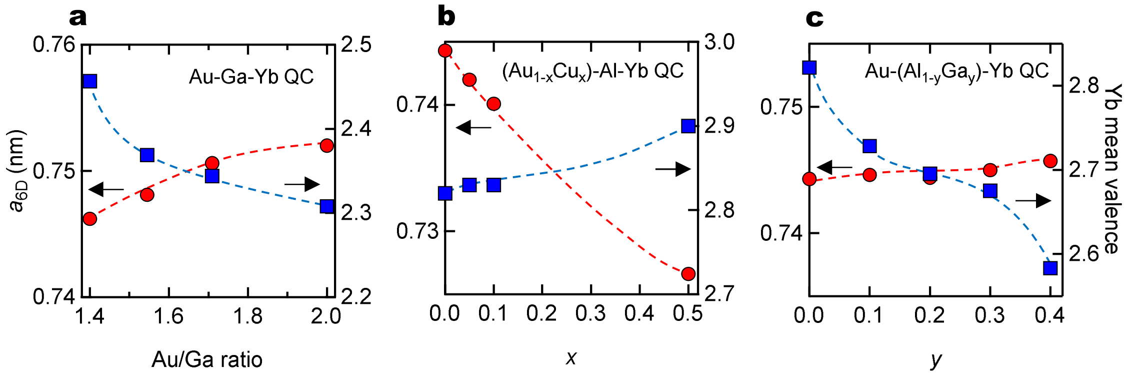

Figure S2 | Substitution effect on the 6D lattice parameter and the Yb mean-valence of QCs. The 6-dimensional lattice parameter a_{6D} and the Yb mean-valence ν are plotted as a function of the constituent element concentration for the Au–Ga–Yb QCs (**a**), the $(Au_{1-x}Cu_x)_{49}Al_{34}Yb_{17}$ QCs (**b**) and the $Au_{49}(Al_{1-y}Ga_y)_{34}Yb_{17}$ QCs (**c**). The closed circles and squares indicate a_{6D} (left axis) and ν (right axis), respectively.

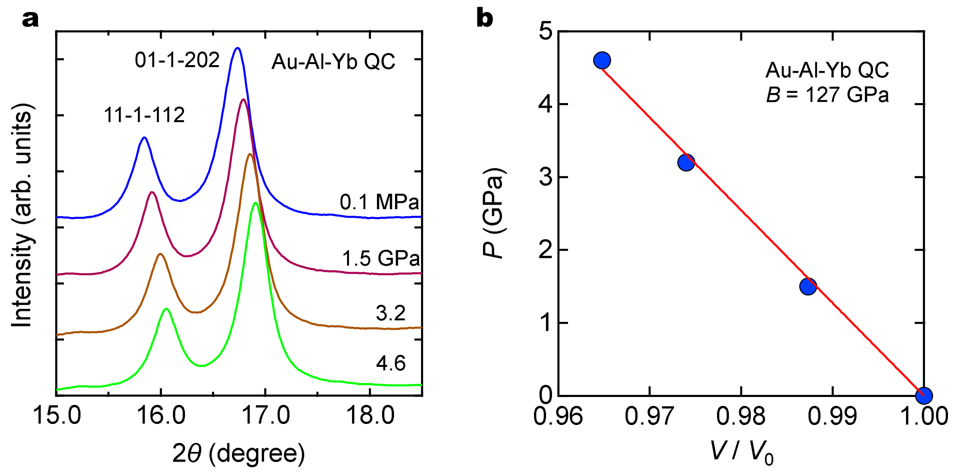

Figure S3 | Pressure dependence of volume of Au–Al–Yb QC. a, Powder XRD patterns at selected pressures below 4.6 GPa around the peaks indexed in the figure. The spectra are shifted vertically for clarity. **b,** Pressure-volume relationship at 300 K. The red solid line shows the fitting result.

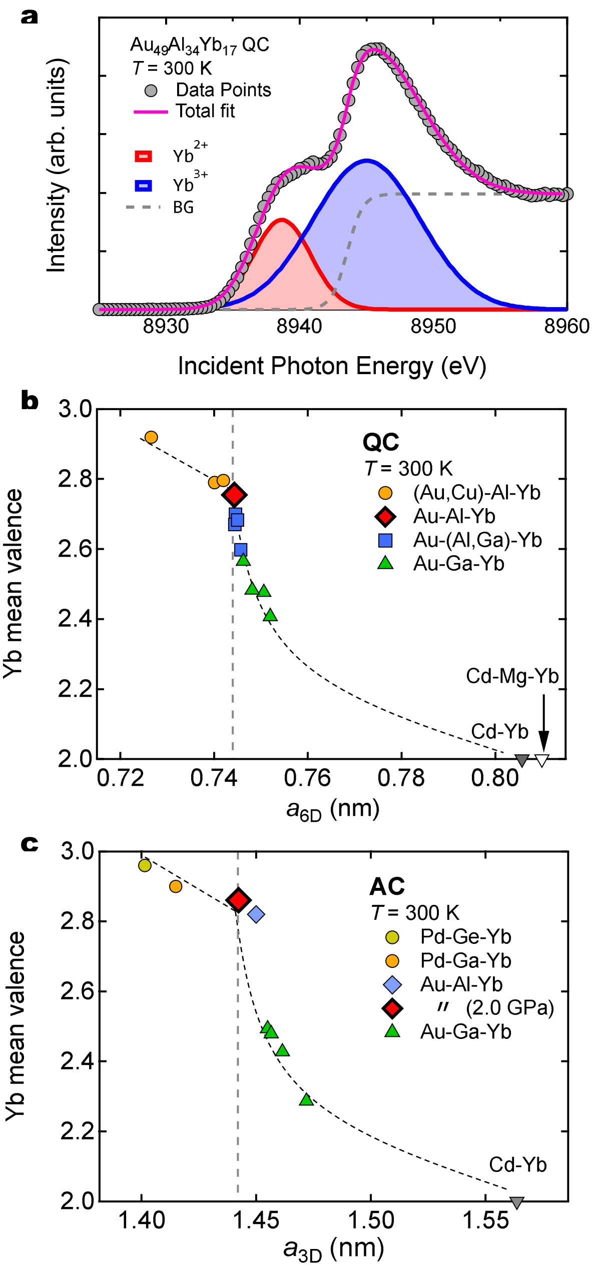

Figure S4 | Lattice parameter dependence of Yb mean-valence. a, Partial-fluorescence-yield x-ray absorption spectra (PFY–XAS) of $Au_{49}Al_{34}Yb_{17}$ QC measured at 300 K. The

double-peak structure is fitted using the double-Gaussian and single-Sigmoid functions for the resonant and fluorescence components, respectively. The red and blue curve are fitted to the resonant component at the L_3 absorption edge of the Yb^{2+} and Yb^{3+} configurations, respectively. **b**. Dependences of Yb mean-valence (ν) on the 6D lattice parameter (a_{6D}). **c**. 3D lattice parameter (a_{3D}) of the Yb-based QCs and ACs. Vertical dashed lines indicate critical lattice parameters $a_{\mathrm{6D}}^{\mathrm{c}}$ = 0.7443 nm of Au–Al–Yb QC and $a_{\mathrm{3D}}^{\mathrm{c}}$ = 1.4423 nm of Au–Al–Yb AC at 2.0 GPa, respectively. The data points for Cd–Yb and Cd–Mg–Yb QCs with Yb^{2+} were taken from Refs. 29 and 30.

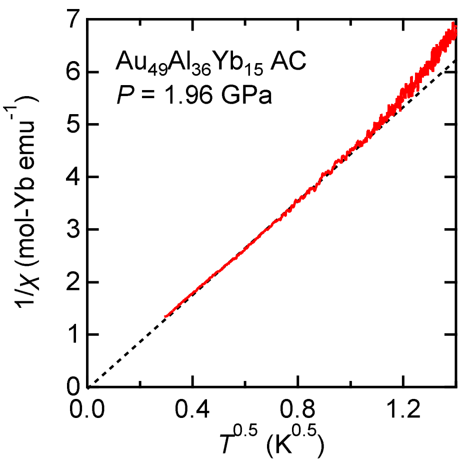

Figure S5 | Ac magnetic susceptibility of Au–Al–Yb AC at the critical pressure (1.96 GPa) below 2 K. The inverse magnetic susceptibility $1/\chi = \frac{\partial H}{\partial M}$ of Au–Al–Yb AC is plotted as a function of $T^{0.5}$.

Table S1 | List of 6-dimensional lattice parameter, Yb mean-valence at 300 K and magnetic susceptibility at 2 K of the QC samples studied here.

Sample	a_{6D} (nm)	Yb valence	χ (emu/mol Yb)	Label in Fig.3
$(Au_{0.5}Cu_{0.5})_{49}Al_{34}Yb_{17}$	0.7266	2.90	4.0×10^{-1}	
$(Au_{0.9}Cu_{0.1})_{49}Al_{34}Yb_{17}$	0.7401	2.83	9.9×10^{-2}	A
$(Au_{0.95}Cu_{0.05})_{49}Al_{34}Yb_{17}$	0.7420	2.83	7.7×10^{-2}	
$Au_{49}Al_{34}Yb_{17}$	0.7443	2.82	5.6×10^{-2}	
$Au_{49}(Al_{0.9}Ga_{0.1})_{34}Yb_{17}$	0.7446	2.73	3.5×10^{-2}	
$Au_{49}(Al_{0.8}Ga_{0.2})_{34}Yb_{17}$	0.7444	2.70	2.3×10^{-2}	
$Au_{49}(Al_{0.7}Ga_{0.3})_{34}Yb_{17}$	0.7450	2.66	1.6×10^{-2}	B
$Au_{49}(Al_{0.5}Ga_{0.5})_{34}Yb_{17}$	0.7457	2.58	9.4×10^{-3}	
$Au_{46.7}Ga_{37.3}Yb_{16}$	0.7461	N/A	9.1×10^{-3}	
$Au_{49}Ga_{35}Yb_{16}$	0.7462	2.46	6.7×10^{-3}	C
$Au_{51}Ga_{33}Yb_{16}$	0.7481	2.37	4.3×10^{-3}	
$Au_{53}Ga_{31}Yb_{16}$	0.7506	2.34	3.3×10^{-3}	
$Au_{56}Ga_{28}Yb_{16}$	0.7520	2.31	2.6×10^{-3}	

Table S2 | List of 3-dimensional lattice parameter, Yb mean-valence at 300 K and magnetic susceptibility at 2 K of the AC samples studied here.

Sample	a_{3D} (nm)	Yb valence	χ (emu/mol Yb)	Label in Fig.3
$Pd_{45.6}Ge_{38.9}Yb_{15.5}$	1.4014	2.96	8.0×10^{-1}	
$Pd_{30}Ga_{55}Yb_{15}$	1.4149	2.87	3.3×10^{-1}	D
$Au_{49}Al_{36}Yb_{15}$	1.4500	2.78	3.8×10^{-2}	E
$Au_{49}Al_{36}Yb_{15}$ (1.96 GPa)	1.4425	N/A	1.5×10^{-1}	
$Au_{49}Al_{36}Yb_{15}$ (2.0 GPa)	1.4423	2.84	N/A	
$Au_{45}Ga_{40}Yb_{15}$	1.4550	2.51	4.1×10^{-3}	F
$Au_{46.7}Ga_{38.3}Yb_{15}$	1.4566	2.49	3.0×10^{-3}	
$Au_{49}Ga_{36}Yb_{15}$	1.4591	N/A	2.2×10^{-3}	
$Au_{51}Ga_{34}Yb_{15}$	1.4614	2.32	9.8×10^{-4}	
$Au_{56}Ga_{29}Yb_{15}$	1.4718	2.16	8.0×10^{-4}	
